# Childbirth, Puerperium and Abortion Care Protocol during the COVID-19 Pandemic

**DOI:** 10.1055/s-0040-1713587

**Published:** 2020-06

**Authors:** Alberto Trapani Júnior, Laura Rassi Vanhoni, Sheila Koettker Silveira, Alessandra Cristina Marcolin

**Affiliations:** 1 Hospital Universitário Professor Polydoro Ernani de São Thiago, Universidade Federal de Santa Catarina, Florianópolis, SC, Brazil; 2 Universidade do Sul de Santa Catarina (Unisul), Palhoça, SC, Brazil; 3 Hospital Regional Homero de Miranda Gomes, São José, SC, Brazil; 4 Hospital das Clínicas, Faculdade de Medicina de Ribeirão Preto, Universidade de São Paulo, Ribeirão Preto, SP, Brazil

**Keywords:** COVID-19, pandemic, pregnancy, guidelines, covid-19, pandemia, gravidez, diretrizes

## Abstract

The new coronavirus (severe acute respiratory syndrome-related coronavirus 2, SARS-CoV-2) is a virus that causes a potentially serious respiratory disease that has spread in several countries, reaching humans in all age groups, including pregnant women. The purpose of this protocol is to provide technical and scientific support to Brazilian obstetricians regarding childbirth, postpartum and abortion care during the pandemic.

## Resumo

O novo coronavírus (*severe acute respiratory syndrome-related coronavirus 2*, SARS-CoV-2) é um vírus que causa uma doença respiratória potencialmente grave que se espalhou por vários países, acometendo seres humanos de todas as faixas etárias, incluindo gestantes. O propósito deste protocolo é fornecer apoio técnico e científico aos obstetras brasileiros com relação aos cuidados no parto, pós-parto e aborto durante a pandemia.

## Introduction

The new coronavirus (severe acute respiratory syndrome-related coronavirus 2, SARS-CoV-2) is a virus identified as the cause of an outbreak of respiratory disease first detected in December 2019 in Wuhan, China.^1^ ^2^ The virus has spread quickly, first throughout Wuhan, and then to other areas of China and other countries in the world. In February 2020, the World Health Organization (WHO) designated the disease as COVID-19, which means coronavirus disease 2019.^1^ ^3^ On March 11, 2020, WHO declared COVID-19 a pandemic disease. 4 On March 20, 2020, Brazilian Ministry of Health (MS, in the Portuguese acronym) recognized the state of community transmission throughout the country's territory.^5^

The measures suggested in this protocol aim to reduce transmission during obstetrical care and control the disease caused by SARS-CoV-2. COVID-19 has a very broad clinical spectrum ranging from no symptoms to severe disease and death. The most common symptoms are fatigue, fever, and dry cough, with more than 80% of hospitalized patients presenting with these symptoms (Box 1). Studies on hospitalized patients show that they commonly develop severe pneumonia, with up to 32% admitted to the intensive care unit (ICU) and between 17% and 29% of the cases progressing to severe acute respiratory syndrome (SARS).^6^

**Box 1:** Clinical findings of patients with COVID-19

More common symptoms	Less common symptoms
• Fever (≥ 37.8°C) • Cough • Dyspnea • Myalgia • Fatigue	• Anosmia or sudden hyposmia • Conjunctival congestion • Anorexia • Sputum production • Odynophagia • Chest pain • Hemoptysis • Dizziness • Headache • Confusion • Nausea/vomiting • Diarrhea • Abdominal pain

Source: Brazilian Ministry of Health.^6^

Given the physiological changes or adverse events that are typical of pregnancy, signs or symptoms related to pregnancy may overlap with other symptoms of COVID-19, thereby making the diagnosis challenging.^7^ The most common complication of the disease is SARS, defined by the onset of dyspnea, decrease in oxygen (O_2_) saturation, signs of respiratory distress or increase in the respiratory rate, worsening clinical conditions of the underlying disease, and arterial hypotension.^7^ In pregnant women, COVID-19 should be considered severe when the patient has at least one or more of the following criteria: respiratory rate ≥ 24 respiratory incursions per minute (ripm), O_2_ saturation < 93%, no improvement in O_2_ saturation with oxygen, hypotension, increased capillary filling time, altered level of consciousness, and reduced urinary output.

### Risk Group

Initially, MS considered as a risk group for COVID-19 individuals over 60 years of age and patients with chronic diseases. Subsequently, the “*Conditions and risk factors to be considered for possible complications of the flu-like syndrome*” were increased to 15, including “*Pregnant women of any gestational age, postpartum women up to two weeks after delivery (including those who had an abortion or fetal loss)*,”^7^ although little evidence indicates that pregnancy makes the patient more susceptible to the new coronavirus than the general population.^6^ ^8^ ^9^ ^10^ On the other hand, it is known that other coronaviruses, which cause Middle East respiratory syndrome (MERS) and SARS, can cause adverse outcomes, increasing maternal and perinatal morbidity and mortality. Considering the various physiological changes, there may be a greater theoretical risk of developing serious COVID-19 during pregnancy.^8^ ^11^ ^12^

Regarding teratogenesis, little or nothing is known about the consequences of SARS-CoV-2, since the literature reports cases of women infected mainly in the second half of pregnancy. To date, there are no reports of congenital anomalies in diseases such as SARS and MERS.^11^ ^13^ However, since any serious infection in pregnancy can harm the fetus,^1^ pregnant woman should avoid professional activities with high risk of contamination or contact with diseased individuals.^14^ Female health professionals who are pregnant and take care of people potentially infected with SARS-CoV-2 should seek the Occupational Medicine Service of their institution for a risk assessment considering the social distancing measures.

### Transmission

The person-to-person transmission of COVID-19 is thought to be similar to the transmission of influenza and other respiratory pathogens. The transmission usually occurs through the air by respiratory droplets, saliva, sneezes, coughs, phlegm, close personal contact (less than 2 m of distance), or through personal contact with contaminated surfaces, touches or handshakes, followed by contact with the mouth, nose or eyes.^10^ ^15^

### Incubation Period

According to information from the Centers for Disease Control and Prevention (CDC), the COVID-19 incubation period varies from 2 to 14 days, and most cases occur within 4 to 5 days.^16^

### Immunity

Preliminary evidence suggests that the antibodies induced by the infection are protective, although this has yet to be confirmed by studies with stronger evidence. Furthermore, it is not known whether all infected patients have a protective immune response and how long this effect lasts.^1^

## Comorbidities in Pregnancy and COVID-19

Given the worse results (including higher mortality) among the population with comorbidities (particularly diabetes and/or hypertension), it is important to consider the potential impact of pre-existing hyperglycemia and hypertension on the outcome of COVID-19 in pregnant women. Currently, there are no studies clarifying this issue; however, it is logical to assume that pregnant women with comorbidities would be at greater risk of presenting serious clinical manifestations based on data from non-pregnant women.

### Clinical Presentation

Retrospective studies involving pregnant women with COVID-19 demonstrated that they have clinical characteristics similar to those of adult non-pregnant women.^17^ ^18^ ^19^ The patients may present fever (with or without chills), cough and/or difficulty breathing. They may also show signs of flu-like syndrome, such as nasal congestion, coryza, anosmia and myalgia. Pulmonary auscultation may reveal inspiratory rales and/or bronchial breathing in pregnant women with pneumonia or pulmonary impairment. Patients with respiratory distress may present tachycardia, tachypnea or cyanosis accompanied by a drop in O_2_ saturation.^6^ ^7^ The physical examination should include an assessment of the respiratory pattern, cough and/or dyspnea; temperature checking; measurement of the heart rate, respiratory rate and pulse oximetry; careful pulmonary auscultation, and evaluation of signs of cyanosis and hypoxia.^6^ ^7^

### Cases will be Considered Suspected if the Patient Presents

1. Flu-like syndrome: acute respiratory condition characterized by a feverish sensation or fever,^1^ even if reported accompanied by cough or sore throat or runny nose or difficulty breathing. Note that fever may not be present in the flu-like syndrome.^6^
2. SARS: flu-like syndrome that presents dyspnea/respiratory discomfort or persistent pressure in the chest or O_2_ saturation lower than 95% in room air, or bluish color of lips or face.^6^

## Cases will be Considered Confirmed if the Patient Presents

1. A flu-like syndrome or SARS with laboratory diagnosis by molecular biology testing (reverse transcription-polymerase chain reaction, RT-PCR) with detection of SARS-CoV-2, influenza or respiratory syncytial virus or immunological testing (rapid test or classic serology for antibody detection), with positive result for immunoglobulin M (IgM) and/or immunoglobulin G (IgG) antibodies in a sample collected after the seventh day of the onset of symptoms.^6^
2. Epidemiological clinical criteria: cases of flu-like syndrome or SARS with history of close personal contact with suspected cases seven days before the onset of symptoms or laboratory-confirmed cases of COVID-19 in which it was not possible to carry out the specific laboratory investigation.^6^

### Laboratory Tests

For diagnostic confirmation, the real-time RT-PCR test for SARS-CoV-2 using material from nasal or oral swabs or nasopharyngeal aspirates is the most conclusive method, and should be performed between the third and seventh days after the onset of symptoms.^6^ The samples must be kept in a refrigerator (at a temperature between 4°C to 8°C) and analyzed within 24 to 48 hours after the collection.

Antibody tests, including IgM and IgG, may be performed for serological evaluation. Immunoglobulin M appears earlier, and it can be detected three to seven days after the onset of symptoms. Thereafter, IgM titers decrease, while IgG titers increase rapidly. Immunoglobulin G titration during the recovery phase can increase by four times or more compared with the acute phase.^6^

In severe cases, some tests should be added: influenza test, pulse oximetry, arterial blood gases (assessment of hypercarbia or acidosis), chest computed tomography (CT), glycemia, urea and creatinine, total and fractions bilirubin, D-dimer, blood count, coagulation tests (prothrombin time [PT] and activated partial thromboplastin time [aPTT]), inflammatory markers (serum procalcitonin and/or C-reactive protein), serum troponin, and serum lactate dehydrogenase. Increased C-reactive protein and lymphopenia are the most common findings.^7^

If radiography and/or chest tomography are considered necessary, they should be performed promptly. Chest radiography may be performed with abdominal protection. The radiation during a chest X-ray (0.0005 mGy to 0.01 mGy) or a chest CT (0.01 mGy to 0.66 mGy) is emitted at doses much lower than those considered teratogenic. The pregnant woman should be informed about the risks and benefits of the exam. When available, pulmonary ultrasound can be a quick alternative for diagnostic complementation.^14^

The imaging exams should not be used as a screening test or for the initial diagnosis of COVID-19. They should be indicated for symptomatic hospitalized patients or in specific clinical situations.^6^ ^7^ Chest X-ray typically shows multifocal airspace opacities in a similar way to other influenza, SARS, MERS and H1N1 infections, and these findings are delayed in comparison with the chest CT. In chest CT, the pulmonary abnormalities of COVID-19 disease are opacities with peripheral, focal or multifocal ground-glass attenuation, and are bilateral in most cases. After 9 to 13 days of the progression of the disease, there is the appearance of lesions with mosaic-paving pattern and consolidations.^2^

## Treatment of COVID-19

To date, we do not have a proven effective treatment for COVID-19 or one that is specific for pregnant women.^7^ ^14^ Symptomatic treatment and pregnancy-specific management of complications such as sepsis and SARS comprise the current standards of care. Differential diagnoses and appropriate clinical management should be taken into account. Oseltamivir phosphate is widely recommended in the early stages of flu in pregnant women.^7^ There have been suggestions to use other drugs in the treatment of COVID-19, such as other antivirals, antibiotics, corticosteroids, serum from healed patients, antimalarials and antiparasitic drugs, but good-quality scientific evidence is still lacking for their use in clinical practice. The use of therapeutic agents should be made after an individual risk-benefit analysis and careful consideration of the potential benefits for the mother and fetal safety.

## Care during Childbirth, Postpartum or Abortion

### Asymptomatic Cases

In asymptomatic patients without flu-like or respiratory symptoms in the previous 14 days, and without close contact with people with flu-like syndrome or suspected SARS-CoV-2 infection within the same period, usual care should be adopted during hospitalization.

### Suspected or Confirmed Cases

First of all, every pregnant woman or parturient and her companion must be screened for suspected or confirmed cases of COVID-19. Suspected or confirmed patients, as well as their companions, should immediately receive a surgical mask that should be changed when it gets wet or after four hours, and they should also be instructed about cough and hand hygiene procedures. In addition, they should stay under contact isolation.^6^ ^7^ Professionals are advised to take precautions in the care of all patients, using appropriate personal protective equipment (PPE) such as surgical masks, gloves, goggles, face shields, surgical caps and aprons. Before and after contact with patients or surfaces close to them, hand hygiene should be performed with 70% alcohol or soap and water. During procedures with production of aerosols, the health team must also use N95/PFF2 masks with face shields.^6^ ^7^ During labor, there must be a continuous assessment of maternal oxygen saturation (SatO_2_ by pulse oximetry) and hourly control of vital signs. If the condition worsens, with fever/hypothermia, dyspnea, difficulty breathing, cyanosis, intercostal runs, respiratory rate > 24 rpm, heart rate > 125 bpm, SatO_2_ < 95%, hypotension, oliguria, leukopenia, thrombocytopenia, irritability, or mental confusion, request an assessment by the intensive care team and implement necessary support measures.^6^ ^7^

### Place of Delivery

To date, no study has shown that delivery in a non-hospital environment is safer because of the pandemic. The Brazilian Federation of Gynecology and Obstetrics Associations (FEBRASGO) reinforces that hospital environment is the most appropriate to reduce maternal and perinatal morbidity and mortality, even in cases of low-risk and asymptomatic pregnant women. Maternity hospitals and other hospitals adopt specific safety and standards of care reduce the risk of contagion. Suspected or confirmed patients with COVID-19 should be admitted to high-complexity referral hospitals in cases of maternal and/or fetal decompensation. Delivery of suspected or confirmed women with COVID-19 is not recommended in households or natural birth centers.

### Presence of Companions, Visitors and Doulas

The goal is to minimize the number of people circulating in the hospital environment. The presence of a companion will be allowed according to the rules of each institution. A maximum of one companion per patient is recommended during the entire hospitalization, and they should be aged between 18 and 59 years, without flu-like symptoms or contact with individuals with flu-like symptoms in the previous 14 days, living at the same household as the parturient, and with no chronic diseases. The companion must wear a surgical mask and be advised about general contact and hygiene care. Due to the recommended social isolation, the presence of doulas, photographers and visitors during hospitalization is not recommended, because the risk of transmission of COVID-19 increases with the number of people circulating in the environment.

### Timing and Mode of Delivery

The timing and mode of delivery should not be determined by the maternal SARS-CoV-2 infection. A multidisciplinary assessment regarding patient's general condition, gestational age and fetal vitality is required. For women with suspected or confirmed COVID-19 in early pregnancy who recover, no change in gestational age at birth is recommended. For women with suspected or confirmed COVID-19 in the third trimester who have not yet recovered, it is reasonable to try to postpone delivery (if maternal and fetal conditions allow it) until obtaining a negative result, in an attempt to prevent transmission to the newborn (NB). In general, COVID-19 infection is not an indication to promote delivery, although early delivery and cesarean section are indicated for pregnant women who develop severe or critical symptoms. In pregnant women with good clinical conditions, mild symptoms and normal fetal well-being, vaginal delivery is safe and recommended.^10^ ^17^ ^18^

### Vertical Transmission

At this time, it is unknown whether SARS-CoV-2 can be transmitted from the mother to the fetus. In a few cases of infected NBs, it was not clear whether transmission was transplacental or postnatal.^20^ ^21^ New evidence suggests that vertical transmission is likely, although the proportion of affected pregnant women and the significance for the NB are still unclear. Two reports published evidence of IgM for SARS-CoV-2 in neonatal serum at birth.^10^ Perinatal infection can cause harmful effects to the conceptus, such as preterm birth, fetal oxygenation disorders, acute respiratory distress, thrombocytopenia accompanied by liver function disorders, and death.^21^ This perinatal risk appears to be independent from the mode of delivery. To date, studies have not demonstrated the presence of the virus in breast milk and amniotic fluid, but have shown them in feces, blood, and maternal urine.^14^ ^17^ ^22^

### Labor Induction

Labor induction can be performed when the pregnant woman is in good clinical condition. During the pandemic period, hospitalization period should be decreased, and the association of induction methods, such as mechanical method and oxytocin, can be offered to patients with previous cesarean section, and mechanical and misoprostol method to patients without a previous cesarean section.^17^

### Operative Delivery

Operative vaginal delivery is not an indication for the parturient just because of the suspicion or confirmation of COVID-19. On the other hand, data available are not sufficient to contraindicate this procedure if it is necessary to ultimate delivery.^12^ ^18^

### Fetal Assessment during Labor

Continuous electronic fetal monitoring is recommended during labor for all cases of suspected or confirmed COVID-19. If it is not possible, follow strict surveillance with intermittent auscultation of the fetal heartbeat every 15 minutes. Changes in the fetal heart rate pattern may be an early indicator of worsening maternal respiratory condition.^6^

### Labor Analgesia and Anesthesia

Axial analgesia should be offered when it is appropriate, and according to the patient's desire. There is no evidence of an increased risk of virus transmission with anesthesia or spinal and/or epidural analgesia. However, general anesthesia should be avoided, as intubation generates aerosols that increase the risk of contagion. Parenteral pharmacological analgesia can be used with caution due to the potential risk of respiratory-center depression.^17^ Non-pharmacological methods of pain relief should be offered, but they cannot be shared with another parturient. The evidence does not support any particular mode of birth, but birthing pools should be avoided when women have suspected or confirmed COVID-19 because of the presence of SARS-CoV-2 in feces, blood, and urine.^23^ ^24^ ^25^ Shower is recommended for hygiene, pain relief and relaxation.^10^

### Intrauterine Resuscitation Measures

Although oxygen via nasal cannula is not an aerosol procedure, the cannula/face mask is in contact with the maternal respiratory tract, and handling the equipment (on/off/adjusting) increases the chance of contagion of the team. Considering the large number of asymptomatic patients and the controversy regarding the possible damages of oxygen therapy, especially when the parturient is not hypoxemic, the need for inhaled oxygen for fetal resuscitation should be carefully analyzed. Other practices that improve uteroplacental perfusion and fetal oxygenation can and should be performed.^12^

### Timely Cord Clamping

Most studies have not demonstrated the presence of SARS-CoV-2 in umbilical-cord blood, either in vaginal birth or cesarean sections. Therefore, we advise waiting 1 to 3 minutes for clamping, since there would be no greater risk of vertical transmission according to the current data.^13^ ^22^ ^26^ ^27^

### Skin to Skin

Skin-to-skin contact is not recommended in patients with COVID-19, as there may be an important contact of the NB with maternal fluids and secretions. The NB should not be positioned in the maternal abdomen or chest.^22^ The NB should be dryed and heated and then bathed.

### Medication Considerations during Pregnancy

Some aspects of medications frequently used during the obstetric care are noteworthy:

- Tocolytics: in pregnant women with suspected or confirmed COVID-19, the tocolytics of choice are nifedipine or atosiban. The indication of tocolysis should be individualized according to the pregnant woman's clinical condition, gestational age, and fetal well-being. Other tocolytics are contraindicated given their potential side effects.^17^
- Prophylactic acetylsalicylic acid: due to the high prevalence of preeclampsia in our population and the reduction of its risk with the use of low-dose aspirin, the prescription of such medication should be maintained in patients with suspected or confirmed COVID-19.^17^ ^18^
- Corticosteroids: the benefits of antenatal corticosteroids for pulmonary maturation in fetuses between 24 and 34 weeks with imminent risk of preterm birth are well established and reduce neonatal morbidity and mortality. Therefore, they must continue to be administered in these situations. Steroids for fetal lung maturation have not been shown to cause more harm in cases of COVID-19. The effects of antenatal corticosteroids for prophylaxis of neonatal morbidity in late prematurity are not yet well established, and they should not be used in patients with suspected or confirmed COVID-19.^17^ ^18^ ^28^
- Prophylaxis for group-B streptococcus: swab collection for the investigation of group B streptococcus should occur as indicated during the usual recommended period, from 35 to 37 weeks of gestation. The use of prophylactic antibiotics to reduce the risk of neonatal sepsis should follow FEBRASGO guidelines.
- Magnesium sulfate: magnesium sulfate should be used according to its usual indications, such as severe hypertension to avoid eclampsia and for the neuroprotection of NBs in extreme prematurity (delivery below 32 weeks), according to FEBRASGO guidelines. There are no data on the impact of magnesium sulfate on COVID-19. However, given the possible respiratory complications with the use of magnesium sulfate, it must be performed carefully in cases with severe respiratory symptoms. Health professionals must be attentive to the signs of intoxication and maintain strict surveillance.^12^
- Prophylactic heparin: evidence suggests the occurrence of microvascular thrombosis in critically-ill patients with COVID-19, although a higher incidence of large-vessel thrombosis has not been demonstrated. Prophylaxis for venous thromboembolism is usually indicated in critically-ill patients.^17^ ^29^ For pregnant women with COVID-19 and other known risk factors for thromboembolic events, the administration of heparin at a prophylactic dose within the already established protocols is recommended.^17^

### Breastfeeding

The benefits of breastfeeding currently outweigh the risks of passing the infection from the mother to the infant. Therefore, breastfeeding is recommended as long as the patient is in good clinical condition. Precautions should be taken, such as: correct hand hygiene; use of a surgical mask by the woman every time she breastfeeds; avoid talking; and rigorous hand hygiene before and between feedings.^10^ ^22^ ^30^ ^31^ If the mother is in the ICU and wishes to breastfeed, her breast milk should be expressed and offered to the NB. In case of severe maternal conditions, care must be taken to avoid breast engorgement. If necessary, milking must be performed.

## Care in Shared Accommodation and Hospital Discharge

Mothers who have tested positive for covid-19 and healthy babies do not require separation. Except for some complications, such as the worsening of maternal health conditions, it is perfectly possible that the mother and child remain together until hospital discharge. However, several precautions must be established. The patient with suspected or confirmed flu-like syndrome and/or COVID-19 must remain in an isolated bed at least two meters away from the NB, wear a surgical mask all the time, which must be changed every two hours and whenever it moistens, and wash her hands frequently and always before touching the NB. It is important that aerosol-generating procedures are not performed in the room, because its increases the risk of contamination.^32^ Hospital discharge must be performed as early as possible, respecting the clinical conditions of the NB and the mother, in 24 and 48 hours after a vaginal delivery and cesarean section respectively.

## Abortion

The cases must be carefully analyzed and individualized so that the patient does not take unnecessary risks when her treatment is postponed, but they should also stay as little as possible in the hospital. In asymptomatic patients with a missed miscarriage, expectant management can be offered for up to four weeks if the patient consents and understands the risks and benefits involved. When necessary, intrauterine aspiration or curettage procedures should be performed, and discharge should be as early as possible.
